# Supplementary material for: Modeling microcephaly with cerebral organoids reveals a WDR62–CEP170–KIF2A pathway promoting cilium disassembly in neural progenitors
Source: Nat Commun. 2019 Jun 13;10:2612. doi: 10.1038/s41467-019-10497-2 (PMC6565620; doi:10.1038/s41467-019-10497-2)
Supplement: Supplementary file 1 — Supplementary Information [file 41467_2019_10497_MOESM1_ESM.pdf]

SUPPLEMENTARY INFORMATION:

Modeling microcephaly with cerebral organoids reveals a WDR62-CEP170-KIF2A pathway promoting cilium disassembly in neural progenitors

Wei Zhang[1,#], Si-Lu Yang[2,#], Mei Yang[1], Stephanie Herrlinger[2], Qiang Shao[1], John L. Collar[2], Edgar Fierro[2], Yanhong Shi[3], Aimin Liu[4], Hui Lu[5], Bruce E. Herring[6], Ming-Lei Guo[7], Shilpa Buch[7], Zhen Zhao[8], Jian Xu[1], Zhipeng Lu[9], and Jian-Fu Chen[1,*]

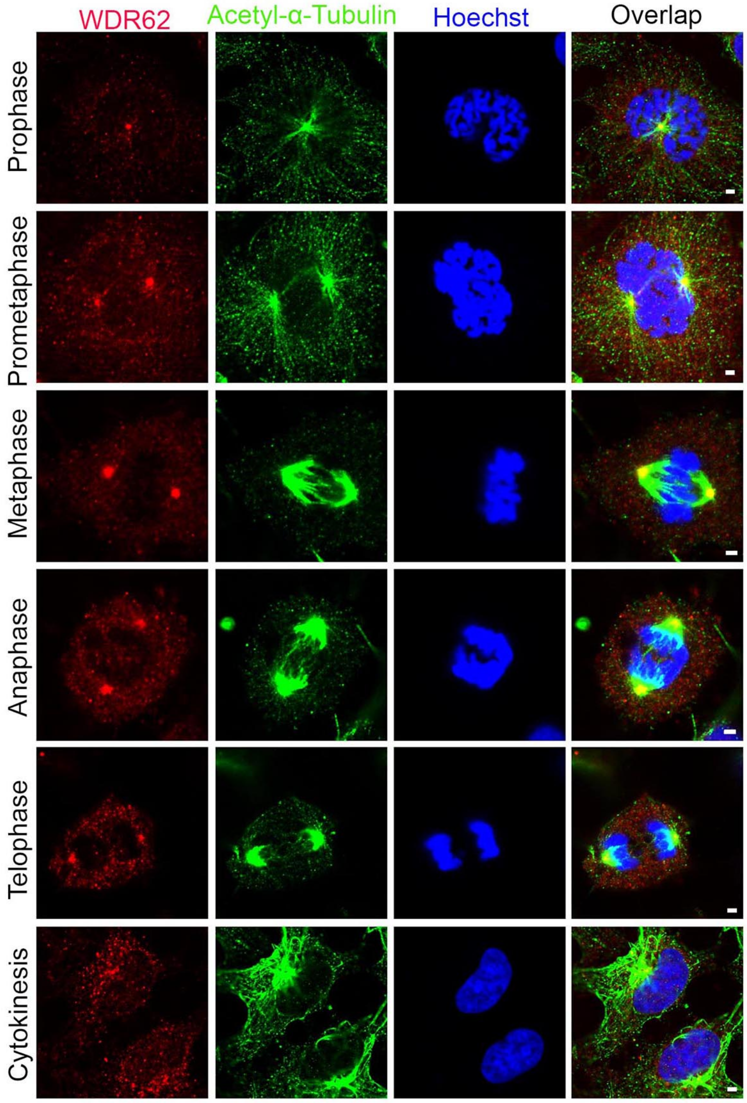

Supplementary Figure 1: WDR62 localization in human NPCs. Confocal imaging of human NPCs at prophase, prometaphase, metaphase, anaphase, telophase, and cytokinesis. Human NPCs were stained with antibodies against WDR62 (red) and acetylated α-tubulin (green). Hoechst stains nuclei (Blue). Scale bars: 2 μm. Note the absence of WDR62 expression in nuclei.

Supplementary Figure 2

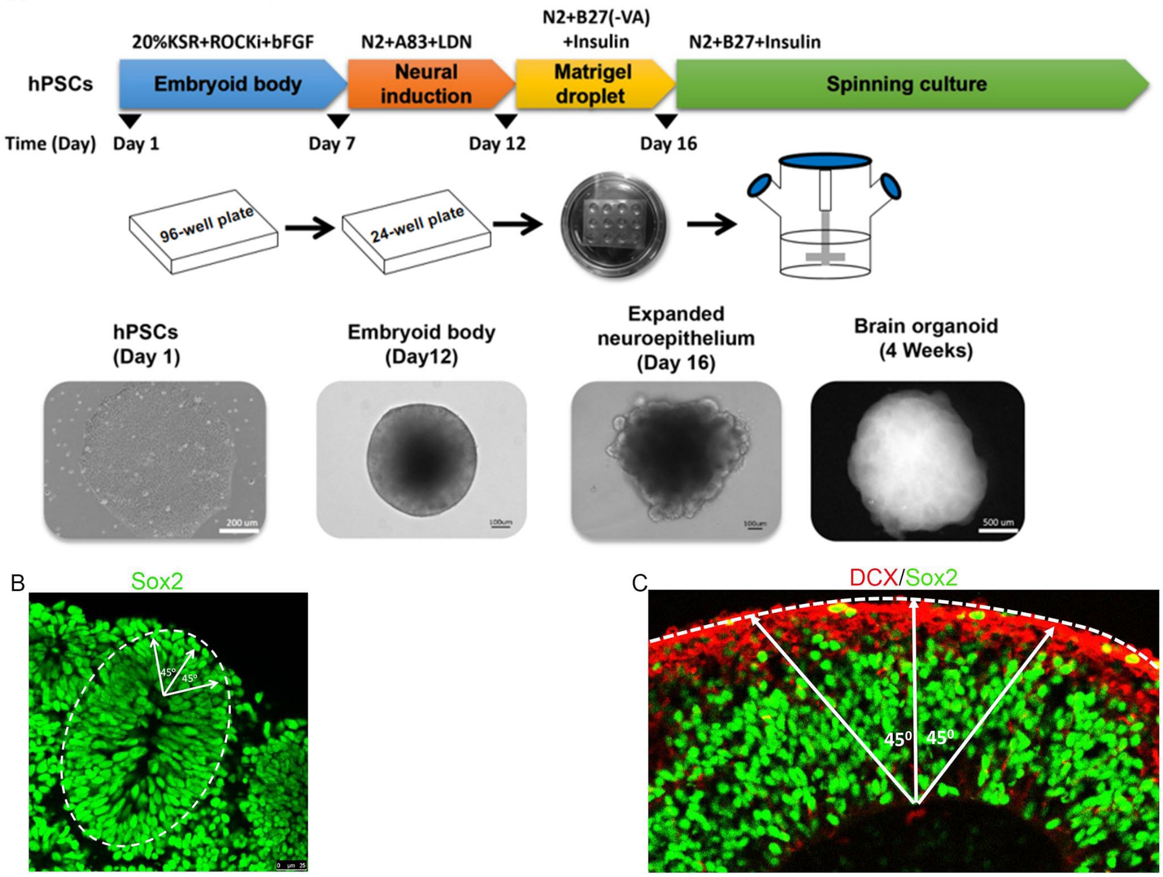

Supplementary Figure 2: Schematic overview of the cerebral organoid culture system and organoid characterization. (A) Schematic diagram of cerebral organoid generation method and representative images for individual stages. hPSCs, human pluripotent stem cells; KSR, knockout serum replacement; ROCKi, ROCK inhibitor; bFGF, basic fibroblast growth factor; A83, A83-01; LDN, LDN-193189; B27(-VA), B27 supplement without Vitamin A. (B) Schematic drawing of $Sox2^+$ (green) ventricular zone (VZ) measurement in cerebral organoids. For each cortical structure, three measurements were taken at 45-degree angles to obtain the mean value. (C) Schematic drawing of $Sox2^+$ (green) VZ and DCX^+ (red) cortical plate (CP) measurement in organoids. For each cortical structure, three measurements were taken at 45-degree angles to obtain the mean value. The relative thickness of DCX^+ neurons was defined as (total layer thickness – VZ layer thickness)/total layer thickness.

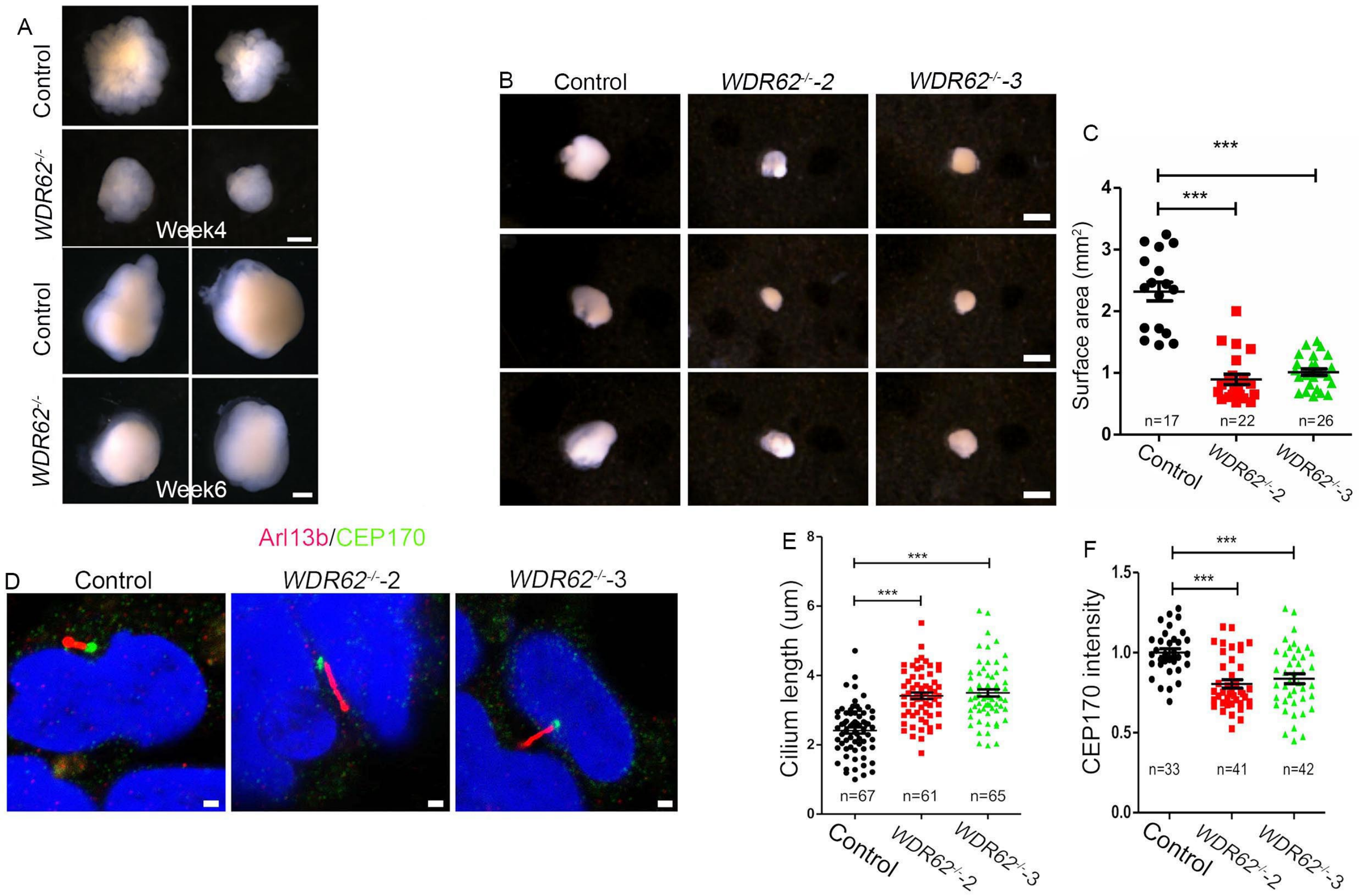

Supplementary Figure 3: Independent *WDR62* mutations resulted in smaller organoid sizes and long cilia. (A) Representative images of control and *WDR62*$^{-/-}$ cerebral organoids at 4 and 6 weeks. Scale bars: 1 mm. (B) Representative images of cerebral organoids from two additional mutant clones with 10bp deletion (*WDR62*$^{-/-}$-2) or 19bp deletion (*WDR62*$^{-/-}$-3) in exon 11. Scale bars: 1 mm. (C) Quantification of surface areas of week 4 cerebral organoids starting with ~5000 cells. Error bars represent SEM of three independent experiments containing numbers of organoids as indicated; ***P < 0.001 (Student's t-test). (D) Confocal imaging of human NPCs stained with antibodies against Arl13b (red) and CEP170 (green). Hoechst stains nuclei. Scale bars: 1 µm. (E) Quantification of cilium length in human NPCs. Error bars represent SEM of three independent experiments from WT (n=67), *WDR62*$^{-/-}$-2 (n=61), and *WDR62*$^{-/-}$-3 (n=65) human NPCs; ***P < 0.001 (Student's t-test). (F) Quantification of CEP170 signal intensity. Error bars represent SEM of three independent experiments containing WT (n=33), *WDR62*$^{-/-}$-2 (n=41), and *WDR62*$^{-/-}$-3 (n=42) human NPCs; ***P < 0.001 (Student's t-test).

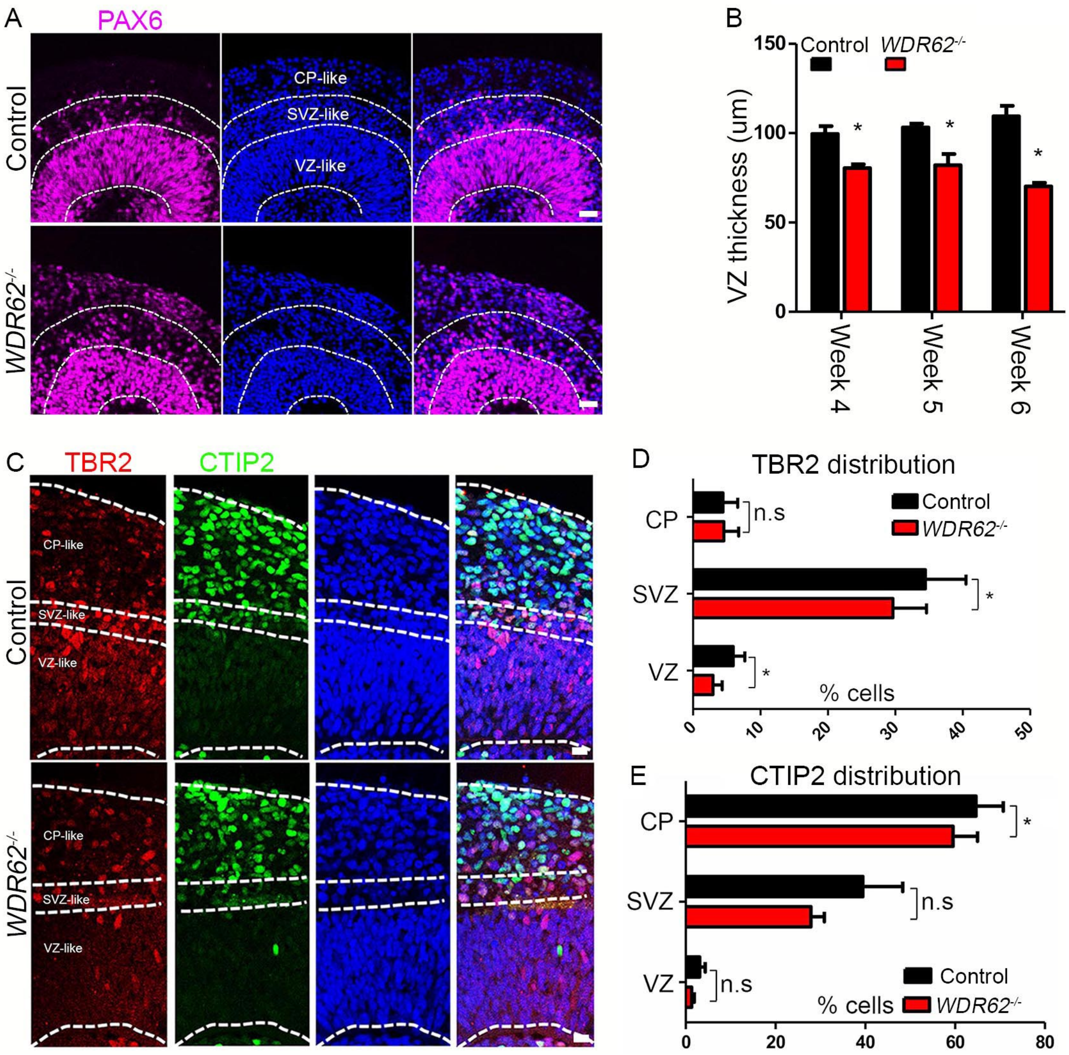

Supplementary Figure 4: Reduced NPCs and CTIP2-positive neurons in mutant organoids. (A) Confocal imaging of sections from week 6 cerebral organoids stained with antibodies against PAX6 (purple). Scale bars: 20 µm. White dotted lines mark ventricular zone (VZ), subventricular zone (SVZ), and cortical plate (CP)-like areas. (B) Quantification of PAX6-positive VZ thickness in 4, 5, or 6 week organoids. Error bars represent SEM of results from four independent cerebral organoids (three sections from each organoid); *P < 0.05 (Student's t-test). VZ thickness was calculated as the mean value derived from three measurements taken at 45-degree angles as measured in Supplementary Figure 2C. (C) Confocal imaging of sections from week 6 cerebral organoids stained with antibodies against TBR2 (red) and CTIP2 (green). Hoechst stains nuclei (blue) Scale bars: 10 um. (D, E) The percentage of TBR2 or CTIP2-positive cells out of total cells within VZ-, SVZ-, and CP-like regions. Error bars represent SEM of three independent experiments (n > 3 sections within each experiments). *P<0.05, n.s represents not significant (Student's t-test).

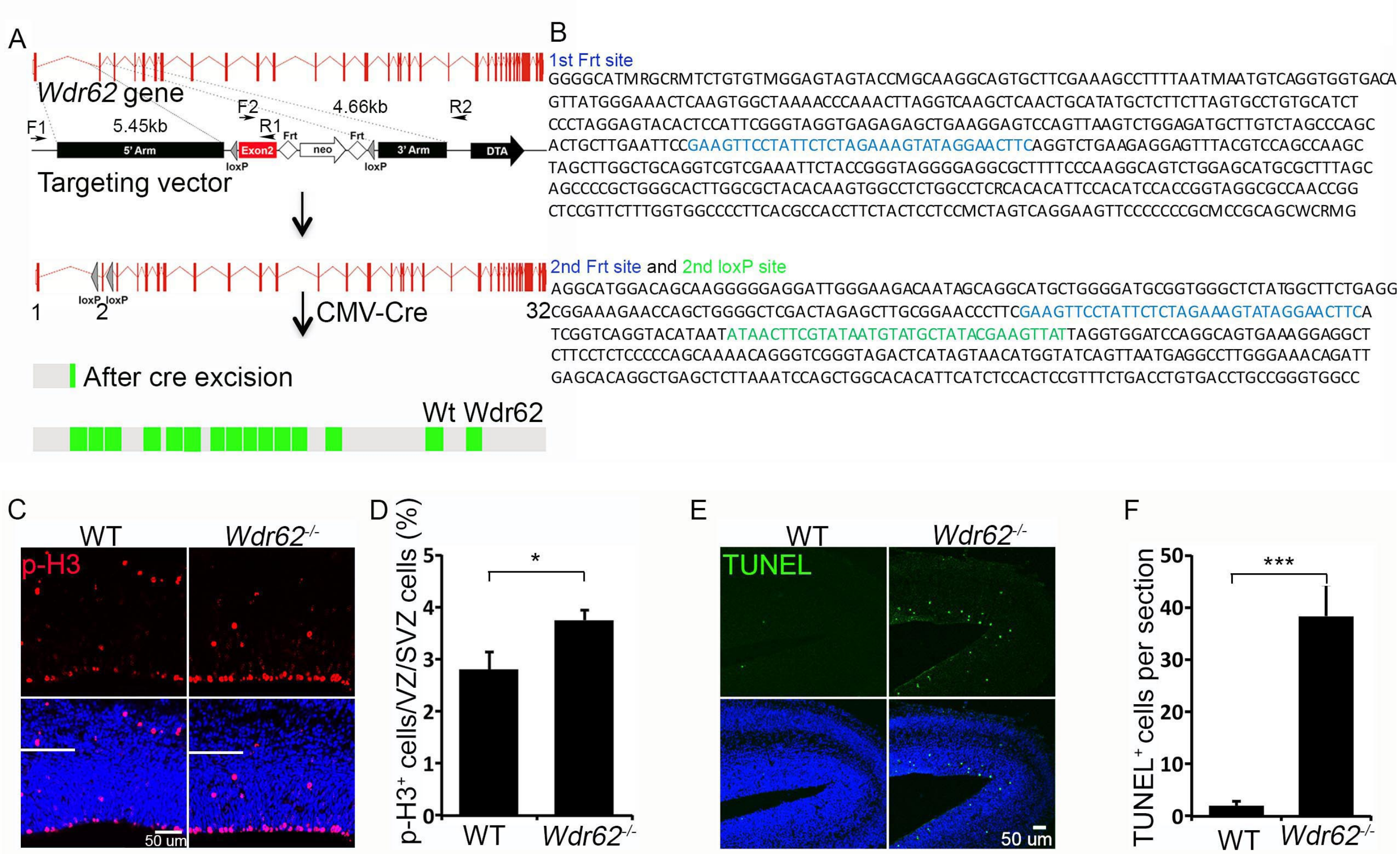

Supplementary Figure 5: Characterization of *Wdr62*$^{-/-}$ mice. (A) Schematic diagram of strategy for *Wdr62* null mouse generation with sequence primers as indicated. (B) Sequence analysis confirmed the Frt and LoxP sites in *WDR62* floxed mouse genome. (C, E) Confocal imaging of coronal sections of E16.5 WT or *Wdr62*$^{-/-}$ cerebral cortex stained with antibodies against p-H3 (red in C) and TUNEL (green in E). Hoechst stains nuclei (blue). Scale bars: 50 μm. Areas below white lines in C represent VZ/SVZ. (D, F) Quantification of the percentage of p-H3-positive cells out of total cells in VZ/SVZ regions (D) or TUNEL-positive cells per sections (F). Error bars represent SEM of three independent experiments with three sections in each experiment; *P < 0.05, ***P < 0.001 (Student's t-test).

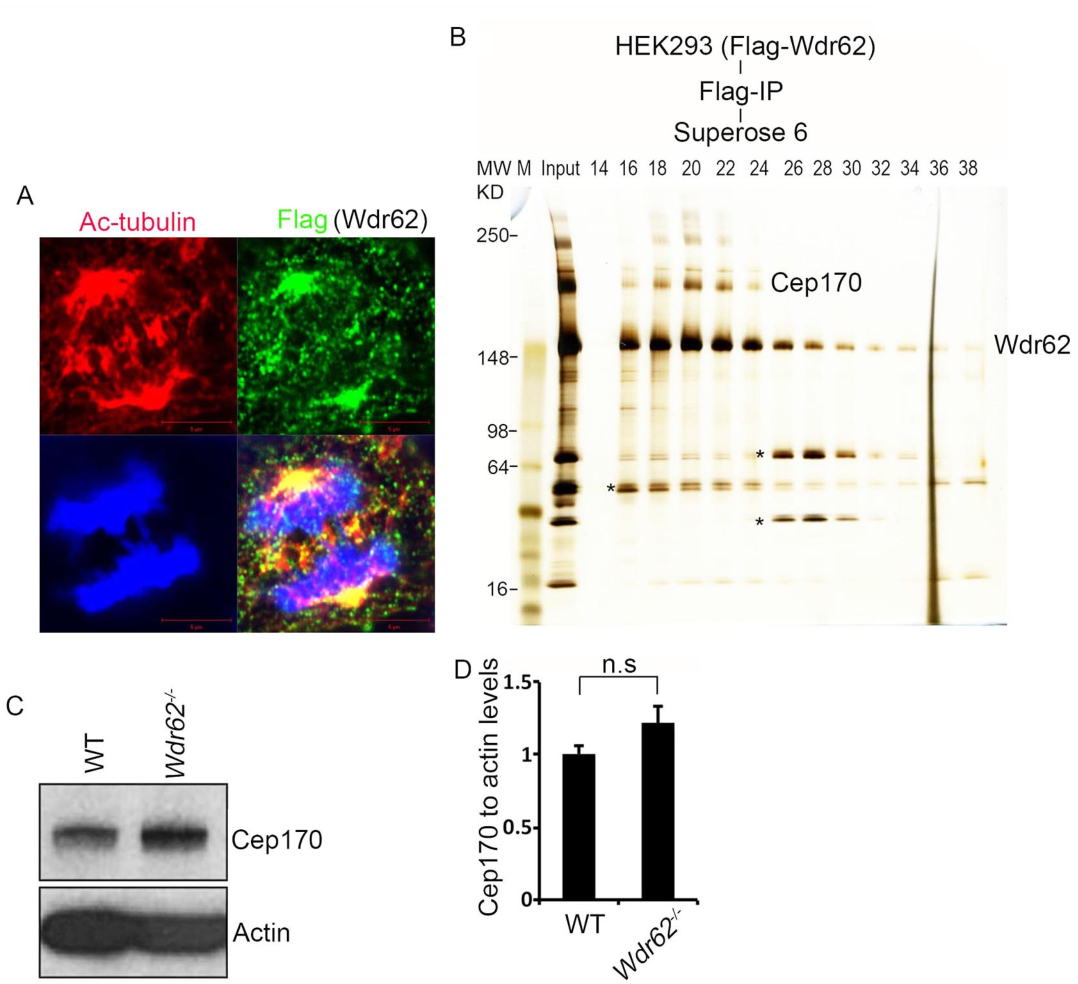

Supplementary Figure 6: Wdr62 interacts with Cep170. (A) Confocal imaging of HeLa cells transfected with Flag-Wdr62 stained with antibodies against acetylated α-tubulin (red) and Flag (green). Hoechst stains nuclei (blue). Scale bars: 5 μm. (B) Superose 6 gel filtration fractions from Wdr62 cytoplasmic flag affinity purification. Fractions were resolved by 4-12% SDS-PAGE and silver stained. The gel filtration purification scheme and fraction numbers are indicated on the top. Asterisks indicate common contaminants of Flag purification (SKB1, α-tubulin and MEP50). (C) Western blot analysis of Cep170 protein expression in WT and *Wdr62* mutant MEFs. Actin serves as the loading control. (D) Quantification of Cep170 expression levels in C. Error bars represent SEM of three independent experiments, n.s represents not significant (Student's t-test).

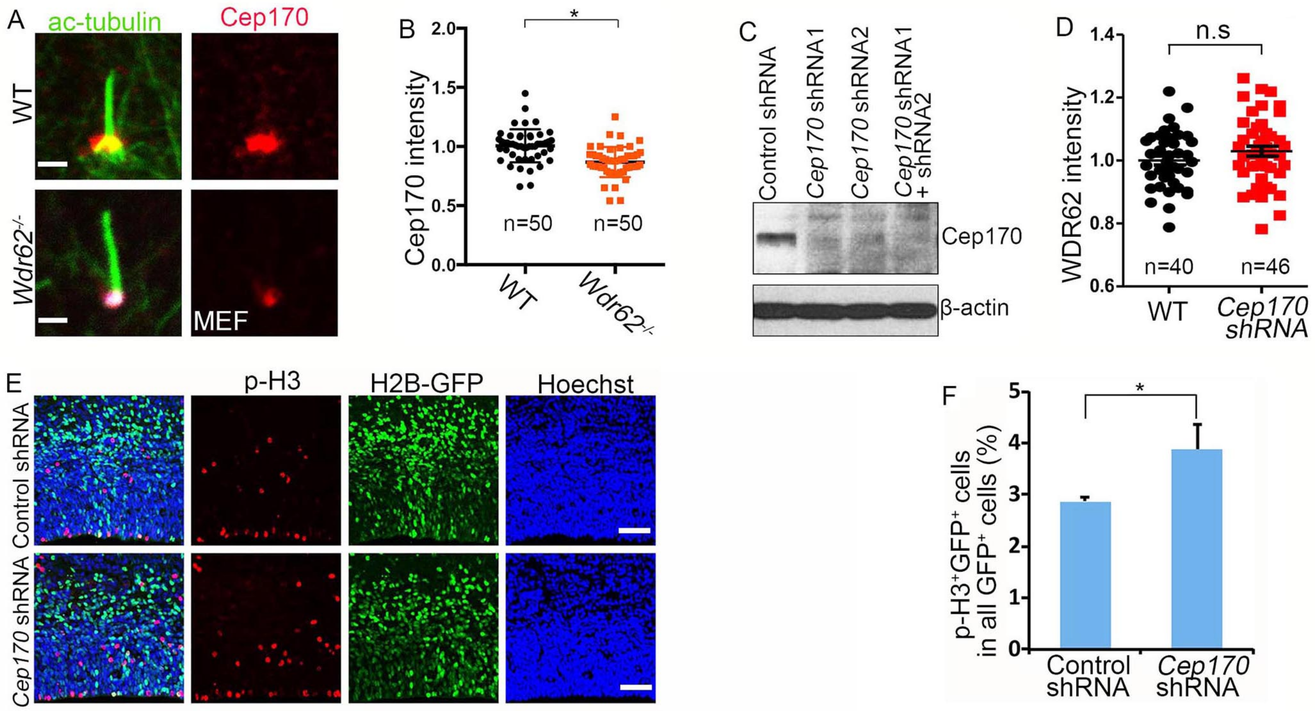

Supplementary Figure 7: Cep170 decreases in *Wdr62* mutant MEFs and its depletion induced mitotic arrest. (A) Confocal micrographs of MEFs stained with antibodies against acetylated α-tubulin (green) and Cep170 (red). Scale bars: 1 μm. (B) Quantification of Cep170 signal intensity in A. Error bars represent SEM of three independent experiments; ~50 ciliated cells were counted in each experiment. *P < 0.05 (Student's t-test). (C) Western blot of Cep170 protein in control and *Cep170* shRNA lentivirus-infected MEFs. β-actin serves as the loading control. (D) Quantification of WDR62 signal intensity at the basal body of the primary cilium. Error bars represent SEM of three independent experiments; n.s represents no significant difference detected (Student's t-test). (E) Confocal imaging of cortex sections stained with antibodies against p-H3 (red) and GFP (green). Hoechst stains nuclei (blue). Scale bars: 50 μm. H2B-GFP and Cep170 shRNA constructs were co-delivered into E14.5 cortex by *in utero* electroporation followed by analysis at E16.5. (F) Quantification of the percentage of p-H3-positive cells out of total GFP-positive cells in the VZ/SVZ areas. Error bars represent SEM of three independent experiments. *P<0.05 (Student's t-test).

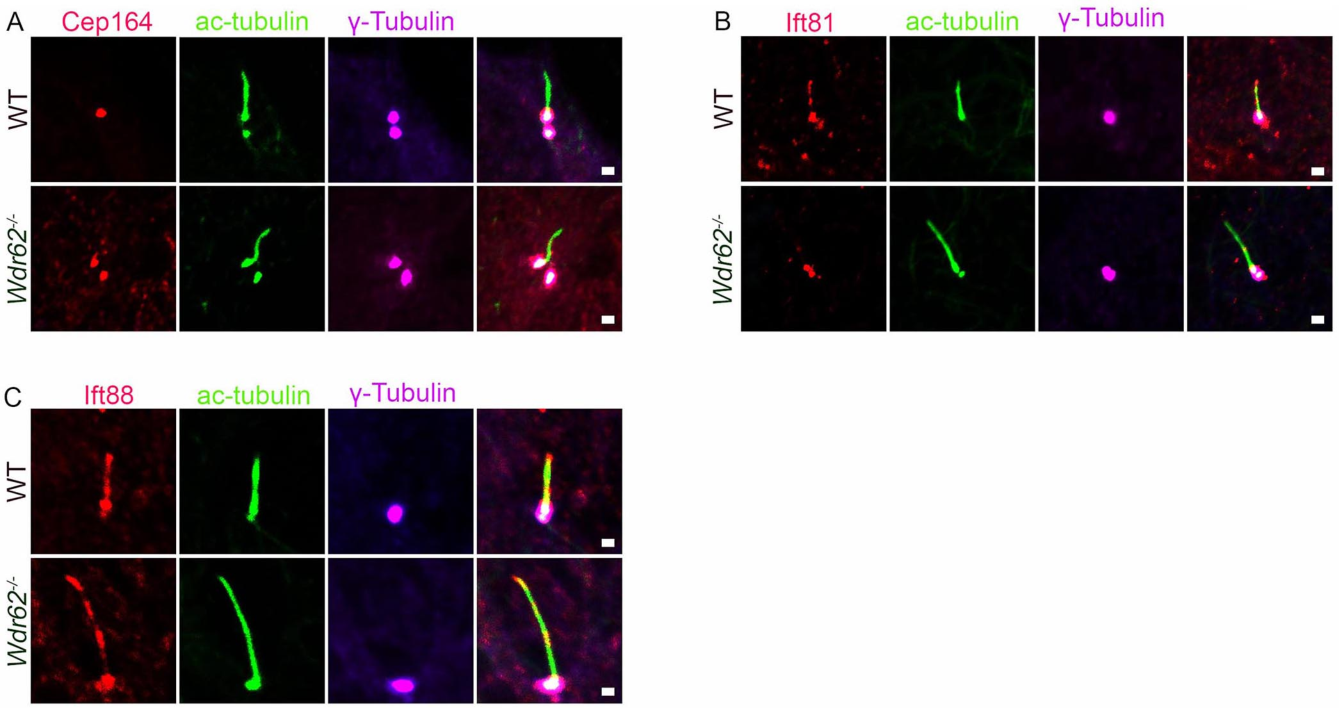

Supplementary Figure 8: Basal body localization of proteins involved in cilium assembly. (A-C) Confocal imaging of WT and *Wdr62* mutant MEFs stained with antibodies against γ-tubulin (purple), acetylated α-tubulin (green), and Cep164 (red in A), Ift81 (red in B), or Ift88 (red in C). Scale bars: 1 μm.

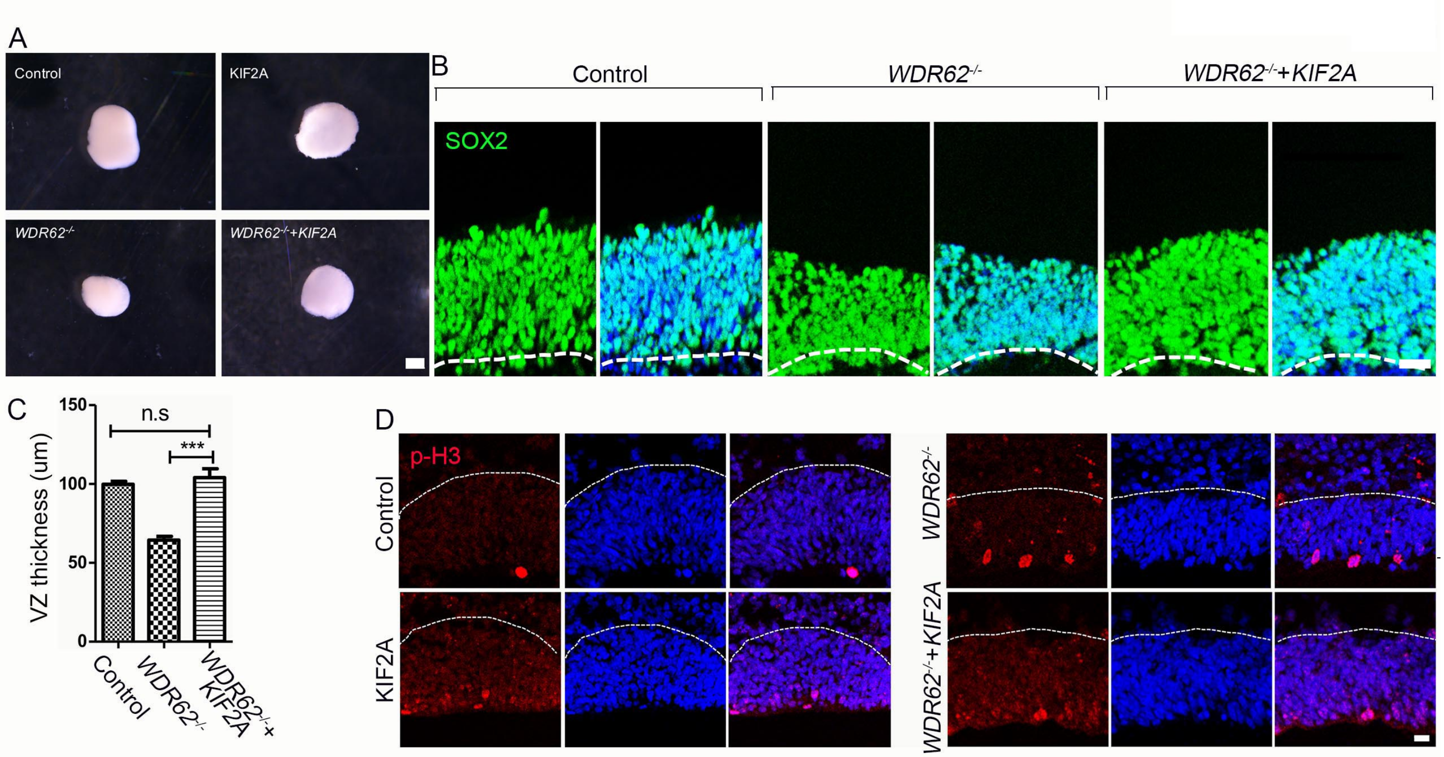

Supplementary Figure 9: Characterization of KIF2A rescue organoids. (A) Representative images of control, *WDR62*$^{-/-}$, and KIF2A rescue cerebral organoids at week 5. Scale bars: 0.5 mm. (B) Confocal imaging of IHC

staining sections from week 5 cerebral organoids. Scale bars: 20 µm. (C) Quantification of SOX2-positive VZ thickness in organoids. Error bars represent SEM of three independent experiments, n.s represents not significant (***P<0.001, Student's t-test). (D) Representative imaging of IHC staining from week 5 cerebral organoids using antibodies against p-H3. Scale bars: 20 µm.

Supplementary Table 1: Primary antibodies used in this study

Name	Company	Catalog	IgG type	Dilution
Anti-Sox2	eBioscience	4294752	Rat IgG	1:500
Anti-Pax6	DSHB	AB_528427	Mouse IgG1	1:200
Anti-Tbr2	Abcam	ab23345	Rabbit	1:200
Anti-Tbr2	R&D	HAF016	Sheep	1:200
Anti-Nestin	eBioscience	14-9843-82	Mouse IgG1	1:1000
Anti-Doublecortin (DCX)	Millipore	AB2253	Guinea Pig IgG	1:5000
Anti-Arl13b	NIH NeuroMab Facility	N295B/66	Mouse IgG2a	1:200
Anti-γ-tubulin	Sigma	T5326	Mouse IgG1	1:1000
Anti-γ-tubulin	Sigma	T5192	Rabbit	1:1000
Anti- Acetylated -tubulin	Abcam	T7451	Mouse IgG2b	1:800
Anti-Kif2a	Abnova	89-115-781	Rabbit	1:1000
Anti-Cep170	Sigma	HPA042151	Rabbit	1:800
Anti-PCM-1	Cell Signaling technology	5213	Rabbit	1:800
Anti-Wdr62	Home made with 1018-1525 aa of Wdr62		Rabbit	1:500
Anti-Brdu	Abcam	ab6326	Rat	1:500
Anti-Wdr62 (For Western blot)	Bethyl Laboratories	A301-560A	Rabbit	1:500
Anti-Ki67	BD Biosciences	550609	Mouse IgG1	1:1000
Anti-PTPRZ1	Sigma	HPA015103	Rabbit	1:500
Anti-Cep164	Dr. Erich A. Nigg	Gift	Rabbit	1:200
Anti-Ift81	Proteintech	11744-1-AP	Rabbit	1:200
Anti-Ift88	Dr. Bradley K. Yoder	Gift	Rabbit	1:200
Anti-KIF24	Dr. Brian David Dynlacht	Gift	Rabbit	1:200
Anti-β3-Tubulin	Cell Signaling technology	5568	Rabbit	1:1000
Anti-p-VIM	MBL international	D076-3	Mouse IgG2b	1:1000
Anti-NF	Abcam	Ab7794	Mouse IG	1:1000
Anti-cleaved Caspase-3	Cell Signaling technology	9661	Rabbit	1:1000
