# Supplementary material for: Modeling microcephaly with cerebral organoids reveals a WDR62–CEP170–KIF2A pathway promoting cilium disassembly in neural progenitors
Source: Nat Commun. 2019 Jun 13;10:2612. doi: 10.1038/s41467-019-10497-2 (PMC6565620; doi:10.1038/s41467-019-10497-2)
Supplement: Supplementary file 3 — Reporting Summary [file 41467_2019_10497_MOESM3_ESM.pdf]

nature research

Corresponding author(s): Jian-Fu Chen

Last updated by author(s): Apr 17, 2019

Reporting Summary

Nature Research wishes to improve the reproducibility of the work that we publish. This form provides structure for consistency and transparency in reporting. For further information on Nature Research policies, see Authors & Referees and the Editorial Policy Checklist.

Statistics

For all statistical analyses, confirm that the following items are present in the figure legend, table legend, main text, or Methods section.

n/a	Confirmed	
☐	☒	The exact sample size (*n*) for each experimental group/condition, given as a discrete number and unit of measurement
☐	☒	A statement on whether measurements were taken from distinct samples or whether the same sample was measured repeatedly
☐	☒	The statistical test(s) used AND whether they are one- or two-sided<br>*Only common tests should be described solely by name; describe more complex techniques in the Methods section.*
☒	☐	A description of all covariates tested
☐	☒	A description of any assumptions or corrections, such as tests of normality and adjustment for multiple comparisons
☐	☒	A full description of the statistical parameters including central tendency (e.g. means) or other basic estimates (e.g. regression coefficient) AND variation (e.g. standard deviation) or associated estimates of uncertainty (e.g. confidence intervals)
☐	☒	For null hypothesis testing, the test statistic (e.g. *F*, *t*, *r*) with confidence intervals, effect sizes, degrees of freedom and *P* value noted<br>*Give P values as exact values whenever suitable.*
☒	☐	For Bayesian analysis, information on the choice of priors and Markov chain Monte Carlo settings
☒	☐	For hierarchical and complex designs, identification of the appropriate level for tests and full reporting of outcomes
☒	☐	Estimates of effect sizes (e.g. Cohen's *d*, Pearson's *r*), indicating how they were calculated

Our web collection on statistics for biologists contains articles on many of the points above.

Software and code

Policy information about availability of computer code

Data collection	*Provide a description of all commercial, open source and custom code used to collect the data in this study, specifying the version used OR state that no software was used.*
Data analysis	Prism 7

For manuscripts utilizing custom algorithms or software that are central to the research but not yet described in published literature, software must be made available to editors/reviewers. We strongly encourage code deposition in a community repository (e.g. GitHub). See the Nature Research guidelines for submitting code & software for further information.

Data

Policy information about availability of data

All manuscripts must include a data availability statement. This statement should provide the following information, where applicable:

- Accession codes, unique identifiers, or web links for publicly available datasets
- A list of figures that have associated raw data
- A description of any restrictions on data availability

All relevant data are available from the corresponding authors upon reasonable request.

Field-specific reporting

Please select the one below that is the best fit for your research. If you are not sure, read the appropriate sections before making your selection.

☒ Life sciences ☐ Behavioural & social sciences ☐ Ecological, evolutionary & environmental sciences

Life sciences study design

All studies must disclose on these points even when the disclosure is negative.

Sample size	We performed a power analysis to determine and confirm sample sizes (N). The values for the power (1-beta) were 0.8 and the type I error rate (alpha) were 0.05.
Data exclusions	We exclude mice based on abnormal health conditions such as weights below 15g at 6 weeks, noticeably reduced activity, or feeding as described in previous studies (Festing, M.F. & Altman, D.G. Guidelines for the design and statistical analysis of experiments using laboratory animals. ILAR J 43, 244-258 (2002)).
Replication	Each experiment will be replicated equal or more than three times . For the statistical analyses, we used three or more than three sets of cultures or tissues from three or more than three animals for each experiment. We described replication and animal numbers in the individual figure legends of the manuscript. Meanwhile, we used different methodologies to confirm the results in each experiment.
Randomization	Individual experiments in this study were performed randomized. We assigned randomly animals to the various experimental groups, collected, and processed data randomly. The handling and treatment of animals were the same across all study groups.
Blinding	Personnel performing and analyzing microscopic imaging results were blinded to genotypes.

Reporting for specific materials, systems and methods

We require information from authors about some types of materials, experimental systems and methods used in many studies. Here, indicate whether each material, system or method listed is relevant to your study. If you are not sure if a list item applies to your research, read the appropriate section before selecting a response.

Materials & experimental systems

n/a	Involved in the study	
☐	☒	Antibodies
☐	☒	Eukaryotic cell lines
☒	☐	Palaeontology
☐	☒	Animals and other organisms
☒	☐	Human research participants
☒	☐	Clinical data

Methods

n/a	Involved in the study	
☒	☐	ChIP-seq
☒	☐	Flow cytometry
☒	☐	MRI-based neuroimaging

Antibodies

Antibodies used	Rat anti-Sox2 (eBioscience, 4294752), Mouse anti-Pax6 (DSHB, AB_528427), Rabbit anti-Tbr2 (Abcam, ab23345), Sheep anti-Tbr2 (R&D, HAF016), Mouse anti-Nestin (eBioscience, 14-9843-82), Pig anti-Doublecortin (DCX) (Millipore, AB2253), Mouse anti-Arl13b (NIH NeuroMab Facility, N295B/66), Mouse anti-γ-tubulin (Sigma, T5326 Mouse), Rabbit anti-γ-tubulin (Sigma, T5192), Mouse anti- Acetylated -tubulin (Abcam, T7451), Rabbit anti-Kif2a (Abnova, 89-115-781), Rabbit anti-Cep170 (Sigma, HPA042151), Rabbit anti-PCM-1 (Cell Signaling technology, 5213), Rabbit anti-Wdr62 (Home made), Rat anti-Brdu (Abcam, ab6326), Rabbit Aanti-Wdr62 (For Western blot, Bethyl Laboratories, A301-560A), Mouse anti-Ki67 (BD Biosciences, 550609), Rabbit anti-PTPRZ1 (Sigma, HPA015103), Rabbit anti-Cep164 (Dr. Erich A. Nigg Gift), Rabbit anti-Ift81 (Proteintech, 11744-1-AP), Rabbit anti-Ift88 (Dr. Bradley K. Yoder Gift), Rabbit anti-KIF24 (Dr. Brian David Dynlacht Gift), Rabbit, anti-β3-Tubulin (Cell Signaling technology, 5568), Mouse anti-p-VIM (MBL international, D076-3), Mouse anti-NF (Abcam, Ab7794), Rabbit anti-cleaved Caspase-3 (Cell Signaling technology, 9661)
Validation	These antibodies were described in the Method section of the manuscript. Validation data are available from the commercial providers. Part of antibodies were also validated in our previous studies.

Eukaryotic cell lines

Policy information about cell lines

Cell line source(s)	The human iPSC line (clone inventory code: R138363028) was obtained from NINDS Human Cell and Data Repository (NHCDR). H9 human ES cells were ordered from the Wisconsin International Stem Cell (WISC) Bank.
Authentication	R138363028) was characterized by NINDS Human Cell and Data Repository (NHCDR). H9 human ES cells were characterized by WiCell Stem Cell Bank.
Mycoplasma contamination	All cell lines were tested negative for mycoplasma

Commonly misidentified lines (See ICLAC register)	N/A

Animals and other organisms

Policy information about studies involving animals; ARRIVE guidelines recommended for reporting animal research

Laboratory animals	Wdr62 knockout mice in C57BL/6N background, males and females at age around 3-4 month-old are included in the study.
Wild animals	N/A
Field-collected samples	N/A
Ethics oversight	All animals were handled according to protocols approved by the Institutional Animal Care and Use Committee at the University of Southern California.

Note that full information on the approval of the study protocol must also be provided in the manuscript.
